# Nonsense mutation in the *CRYBB2* gene causing autosomal dominant progressive polymorphic congenital coronary cataracts

**Published:** 2008-04-24

**Authors:** Fei-feng Li, Si-quan Zhu, Shu-zhen Wang, Chang Gao, Shang-zhi Huang, Meng Zhang, Xu Ma

**Affiliations:** 1 Graduate School, Peking Union Medical College, Beijing, China; 2 Department of Genetics, National Research Institute for Family Planning, Beijing, China; 3 Beijing Tongren Eye Center, Capital Medical University, Beijing, China; 4 WHO Collaborative Center for Research in Human Reproduction, Beijing, China.

## Abstract

**Purpose:**

We sought to identify the genetic defect in a large, five-generation Chinese family with autosomal dominant progressive polymorphic congenital coronary cataracts and to examine the clinical features in detail.

**Methods:**

Clinical and ophthalmologic examinations were conducted on family members. All members were genotyped with microsatellite markers at loci previously associated with cataracts. Two-point LOD scores were calculated using a linkage package after genotyping. A mutation was detected by direct sequencing and verified by denaturing high-performance liquid chromatography (DHPLC).

**Results:**

Clinical observations showed that all affected family members had progressive polymorphic coronary cataracts. Linkage analysis was obtained at markers, D22S303 (LOD score [Z]=2.11, recombination fraction [θ]=0.0) and D22S1167 (Z=1.20, θ=0.0). Haplotype analysis indicated that the cataract gene was closely linked with these two markers. Sequencing the βB-crystallin gene (*CRYBB2*) revealed a C → T transition in exon 6, which changed a codon from Gln to a stop codon (P.Q155X). This mutation cosegregated with all affected individuals and was not observed in any unaffected family member or 100 normal, unrelated individuals.

**Conclusions:**

This study identified a mutation in *CRYBB2* in a large Chinese family with autosomal dominant progressive polymorphic congenital coronary cataracts. These results provide evidence that *CRYBB2* is a pathogenic gene for congenital cataracts; at the same time, congenital cataracts are a clinically and genetically heterogeneous lens condition.

## Introduction

Cataracts are an opacity of the lens that leads to loss of vision, and even blindness, and can be congenital or acquired, unilateral or bilateral [1]. Idiopathic, hereditary syndromes (Down syndrome and Rubinstein-Taybi syndrome), and intrauterine infections (congenital measles) can cause congenital cataracts, and traumatic, metabolism (high blood pressure), and some substances (alcohol and smoking) can cause acquired cataracts [2-4].

Congenital cataracts are a clinically and genetically heterogeneous lens condition responsible for a significant proportion of childhood visual impairment and blindness [5,6]. They can occur in an isolated fashion or as a component of a multi-system disorder. Non-syndromic congenital cataracts have an estimated incidence of 1–6 per 10,000 live births [7-10]. Although congenital cataracts are much less common than age-related cataracts, they are still responsible for approximately 10% of childhood blindness worldwide [11].

Since the first description of the cosegregation of inherited cataracts with the Duffy blood group locus, more than 30 loci have been mapped through linkage analysis and 17 genes have been characterized [12,13]. These genes can be considered in five groups, ten genes encoding crystallins (*CRYAA*, *CRYAB*, *CRYBA1/A3*, *CRYBA*, *CRYBB1*, *CRYBB2*, *CRYBB3*, *CRYGC*, *CRYGD*, and the *CRYGs*), three encoding membrane transport proteins (*MIP*, *GJA3*, and *GJA8*), one encoding cytoskeletal proteins (*BSFP2*), three encoding transcription factors (*HSF4*, *MAF*, and *PITX3*), and one encoding a lens intrinsic membrane protein [14-16].

Len crystallins are subdivided into α, β, and γ crystallins and constitute more than 80%–90% of the water-soluble structural proteins present in the vertebrate crystallin lens [17]. α-Crystallins are heat shock proteins that function as molecular chaperones while γ- and β-crystallins are members of a superfamily of microbial stress proteins, which share a common structural feature of four ‘Greek key’ motifs, two (1 and 2) in the NH_2_- and two (3 and 4) in the COOH-terminal domain [18]. Modifications of crystallins may disrupt their normal structure in the lens and cause cataracts [19].

**Figure 1:**
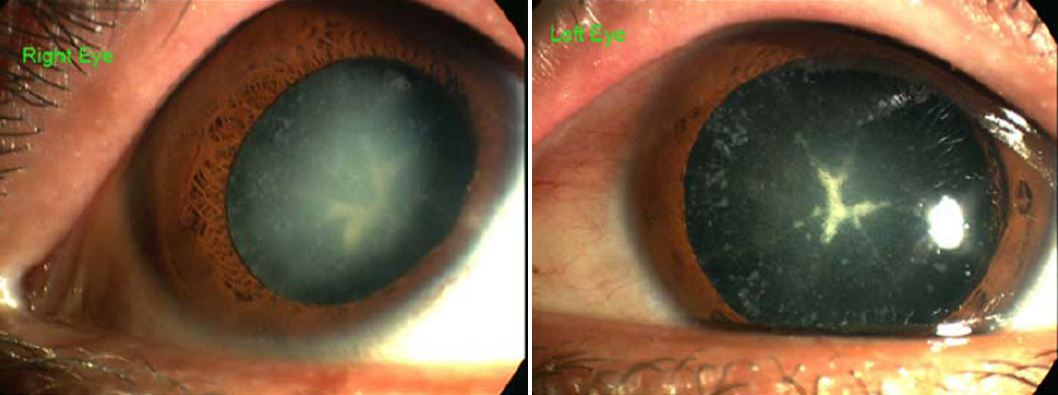
Slit lamp photographs of an affected individual (III:23). The photographs of the affected individual III:23 showed that the opacities were coronary cataracts with punctate, asteroidal, and nuclear opacities. There were pulverulent opacities in the perinuclear areas of the right lens while pulverulent opacities in the left lens were lighter. They were also more coronal in the left eye.

Here, we report a large, five-generation Chinese family with autosomal dominant progressive polymorphic congenital coronary cataracts. Linkage analysis mapped the disease gene to 22q11.2–12.2, and a nonsense mutation (475C → T) in *CRYBB2* was identified in this family, resulting in the substitution of a codon for the conserved amino acid, Gln, with a stop codon. Clinical and ophthalmologic examinations were conducted on family members in detail; all affected members show different clinical features.

## Methods

### Clinical evaluation and DNA specimens

A large, five-generation family with non-syndromic progressive polymorphic congenital coronary cataracts was recruited at the Beijing Tongren Eye Center, Capital Medical University, Beijing, China. Informed consent was obtained from each participant, consistent with the Declaration of Helsinki. The phenotype was documented by slit-lamp photography. Genomic DNA was extracted from peripheral blood leukocytes using standard protocols.

### Genotyping

Polymerase chain reactions (PCRs) were performed with microsatellite markers close to candidate loci associated with autosomal congenital cataracts. PCR products from each DNA sample were separated on a 6% polyacrylamide gel and analyzed. Pedigree and haplotype data were managed using the Cyrillic software (version 2.1). Exclusion analysis was performed by allele sharing in affected individuals [20].

**Table 1:** Clinical features of affected family members.

**Number**	**Age**	**Gender**	**Onset age**	**Surgery age**	**Clinical features**
II1	73	Female	20	30	Aphakia, after cataract surgery
II3	61	Male	14–15	30	Aphakia, after cataract surgery
III1	53	Male	30		Bilateral coronary,punctate, asteroidal (above the posterior pole) cataracts
III2	50	Male	20		Bilateral coronary, punctate cataracts; gray opacity in partial coronary area
III3	42	Female	15	25	Aphakia, after cataract surgery
III4	47	Female	16–17	32	Right coronary, punctate, asteroidal (posterior pole) cataracts; left after cataract surgery
III5	36	Female	14–15	27	Aphakia, after cataract surgery
III20	40	Female	13	22	After bilateral phacoemulsification and intraocular lens implantation
III21	37	Female	10	29	After bialateral phacoemulsification and intraocular lens implantation
III22	35	Male	12		Bilateral punctate, asteroidal (above the posterior pole) cataracts
III23	33	Male	12	33	Proband
IV1	25	Male	12		Bilateral punctate, asteroidal (above the posterior pole) cataracts
IV4	22	Female	12		Bilateral punctate, asteroidal (above the posterior pole) cataracts
IV8	19	Male	10		Bilateral coronary, punctate cataracts
IV10	24	Female	15		Bilateral coronary, inverted T-shaped (posterior pole) cataracts
IV15	13	Male			Bilateral sparse punctate, asteroidal (above the posterior pole) cataracts
IV16	6	Male			Sparse punctate opacities

Before the age of 10, no clinical features were manifest (IV16); punctate opacification was primarily scattered in the lens perimeter, causing almost no influence on the life of the affected individuals. In adolescence, affected individuals showed ambiguous visual clinical features. At about the age of 30, the clinical features became serious. Two affected family members (III4, III23) had different clinical features between their two lenses; III1, III2, III22, and IV10 had different clinical features from each other. The clinical features of III22, IV1, and IV4 were similar.

### Linkage analysis

A two-point linkage was calculated with the LINKAGE package (version 5.1). Autosomal dominant cataracts were analyzed with full penetrance and a gene frequency of 0.001. The allele frequencies for each marker were assumed to be equal in both genders. The marker order and distances between the markers were taken from the NCBI and GDB databases.

### DNA sequencing

Individual exons of the β-crystallin gene cluster were amplified by PCR using primer pairs [21]. PCR products were sequenced using an ABI3730 Automated Sequencer (PE Biosystems, Foster City, CA).

**Figure 2:**
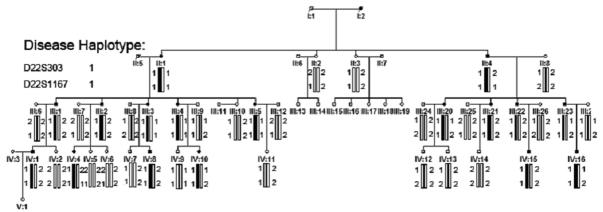
Pedigree and haplotype of the cataract family. Five-generation pedigree segregates autosomal dominant progressive polymorphic coronary cataracts. Haplotyping showed segregation with two microsatellite markers on 22q. Squares and circles indicate males and females, respectively. Blackened symbols and bars denote affected status.

### Denaturing high-performance liquid chromatography

Denaturing high-performance liquid chromatography (DHPLC) was used to screen the mutation identified in affected patients, other family members, and 100 normal control subjects in exon 6 of *CRYBB2* using a commercial system (Wave DHPLC; Transgenomic, San Jose, CA).

## Results

### Clinical data

The proband was a 33-year-old male (III: 23) who had bilateral cataracts. From the age of 12 or 13, he had light apprehension and ambiguous visual clinical features. The condition became serious at the age of 25. Slit-lamp examination (III: 23) showed grayish/bluish punctate opacification in the cortex. A large number of spindle-shaped and oval punctate opacities were directed radially in the periphery, just like coronal cataracts. The clinical features of the left and right lenses showed some differences. No systemic or other ocular anomalies were observed in the patient.

This five-generation family included 17 affected individuals with congenital special-type coronary cataracts (Figure 1) and 34 unaffected individuals. The diagnosis was confirmed by ophthalmologists. The clinical diagnosis of the family was progressive polymorphic coronary cataracts with punctate, asteroidal, and nuclear opacities. Each of the affected individuals showed a somewhat different phenotype; in some affected subjects, star-like opacification was present in the upper side of the posterior pole (Table 1). There was no history of other ocular or systemic abnormalities in the family.

**Figure 3:**
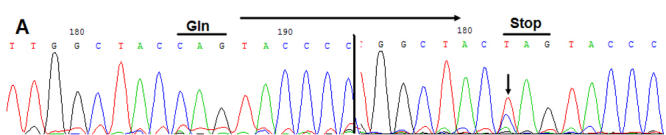
DNA sequence chromatograms of the P.Q155Χ mutation in *CRYBB2*. Forward sequence analysis of the normal and affected sequence of exon 6 of the *CRYBB2* gene in this family is shown. The C→T transition at position 475 resulted in the P.Q155Χ mutation.

### Linkage and haplotype analysis

The *CRYBB2* gene on chromosome 22 was linked to this family’s disease while other candidate genes were excluded by allele sharing and linkage analysis. Significant linkage was found with markers, D22S303 and D22S1167; the maximum LOD score was 2.11 (at θ=0). Haplotype analysis showed that the phenotype was localized at chromosome 22q11.2–12.2 flanked by markers, D22S303 and D22S1167 (Figure 2, Table 2).

### Mutation analysis for *CRYBB1*, *CRYBB2*, *CRYBB3*, and *CRYBA4*

Direct cycle sequencing of the amplified fragments of *CRYBB2* in two affected individuals identified a single base alteration, C.C475T (Figure 3A), in exon 6 of *CRYBB2* (NM_000496), resulting in a substitution of a Gln codon with a stop codon (P.Q155X). The remainder of the coding sequence showed no other change.

**Table 2:** Two-point LOD scores for linkage between cataract locus and chromosome 22 markers

**Marker**	**LOD scores by recombination fraction (θ)**						
	0	0.04	0.09	0.14	0.19	0.24	0.29
D22S303	2.11	1.98	1.82	1.65	1.47	1.27	1.07
D22S1167	1.2	1.13	1.04	0.94	0.84	0.73	0.61

The highest observed LOD score was 2.11 (θ=0) for marker D22S303.

### Denaturing HPLC

**Figure 4:**
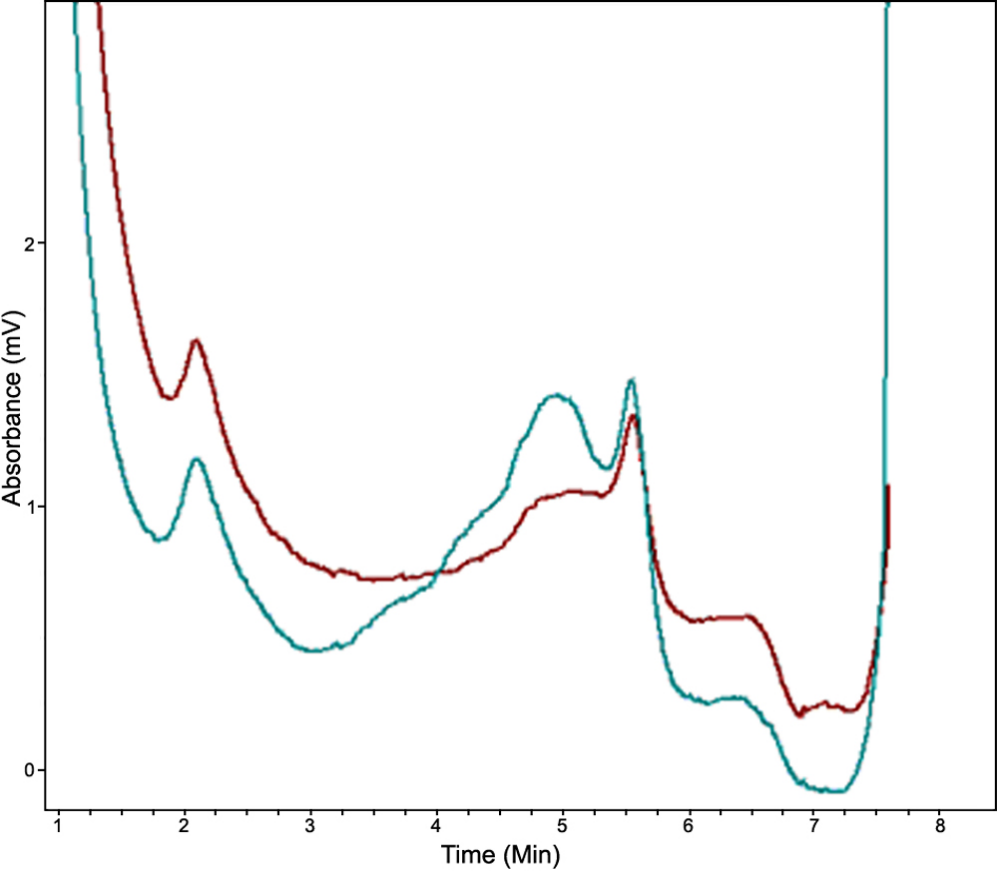
Denaturing high-performance liquid chromatography results of wild type and mutated CRYBB2. DHPLC results show variant traces for CRYBB2 compared with the wild type (WT) trace. The profile in blue is the mutant protein; the profile in red is the wild type protein.

Denaturing HPLC analysis confirmed this mutation (Figure 4), which cosegregated with all affected individuals in the family. Further, this mutation was not observed in any of the unaffected family members or the 100 normal controls.

## Discussion

We identified a mutation, P.Q155X, in *CRYBB2* in a large, five-generation Chinese family with autosomal dominant progressive polymorphic congenital coronary cataracts. The disease gene was linked to 22q11.2–12.2 with a maximum LOD score of 2.11 spanning the β-crystallin gene cluster, which includes *CRYBB1*, *CRYBB2*, *CRYBB3*, and *CRYBA4*. Mutation analysis of the candidate gene detected a mutation, P.Q155X, in *CRYBB2* that cosegregated with the disease phenotype in all affected individuals but was not present in the unaffected family members or the 100 normal control subjects. Clinical and ophthalmologic examinations were conducted on family members in detail; all affected members show different clinical features, and some affected members show different clinical features between the two lenses.

Development of the lens is intrinsically linked to the development of the anterior segment; a transcriptional cascade is involved in early lens development, through Pax6 expression followed by expression of *Mafs, Soxs*, and *Prox1*, resulting in the initiation of lens cell differentiation and crystallin expression [22-27]. The β-crystallins are major constituents of the mammalian lens where they associate into dimers, tetramers, and higher oligomers. The appropriate association of crystallins into higher-order complexes is critical to the maintenance of lens transparency and a high refractive index [28]. Opacification in the lens can hinder light from focusing on the retina during key stages of development and lead to permanent visual impairment [29].

*CRYBB2* consists of six exons encoding a predicted 205 amino acid protein. The first exon is not translated, the second exon encodes the NH_2_-terminal extension, and the subsequent four exons are responsible for one ‘Greek key’ motif each [30]. The α-, β-, and γ-crystallins form heterogeneous oligomers in the lens and have molecular weights ranging from 40 to 200 kDa [31]. So far, there have been eight reports in the literature of *CRYBB2* gene mutations causing congenital cataracts [32-39]. In addition to Pauli et al. [32], who reported a German family with a mutation in exon5 (p.D128V) which caused a nuclear cataract with an additional ring-shaped cortical opacity and Yao et al. [35] who reported a Chinese family with a mutation in exon 6 (p.Q155 X) which caused highly variable white opacities distributed in the nucleus and cortex, which included pulverulent, dot, strip, star-like and sheet shapes, the clinical features of the other reports were relatively simple (include sutural cataracts, cerulean cataracts and Coppock-like cataracts). This is the sixth reported case of a C.C475T mutation causing congenital cataracts, providing further evidence that *CRYBB2* is a pathogenic gene for congenital cataracts and that this site is a hot spot for *CRYBB2* mutation.

The clinical diagnosis in the family was coronary cataracts with punctate, asteroidal, and nuclear opacities. Opacification of the lens in affected individuals was bilateral. Each of the affected individuals showed a somewhat different phenotype. In some affected subjects, the star-like opacifications were present at different sites in the posterior pole. Some affected like the proband (pulverulent opacities in the perinuclear areas of the right lens while pulverulent opacities in the left lens were lighter, and in the left lens, they were more coronal) show different clinical features between the two lenses.

Before the age of 10, no clinical features were manifest in the affected individuals, although there was punctate opacification scattered mainly in the lens perimeter, but this had almost no effect on their lives. In adolescence, affected individuals showed ambiguous visual clinical features, and at about the age of 30, the clinical features became serious. Each of the affected individuals showed a somewhat different phenotype. In some affected subjects, the star-like opacifications were present in the upper side of the posterior pole. The opacification style in each affected individual was not alike; some were star-like and some were of an inverted T-shape. These observations provide evidence that congenital cataracts are a clinically heterogeneous lens condition.
